# Revisiting the Role of Thiopurines in Inflammatory Bowel Disease Through Pharmacogenomics and Use of Novel Methods for Therapeutic Drug Monitoring

**DOI:** 10.3389/fphar.2018.01107

**Published:** 2018-10-08

**Authors:** Sheng Zhang Lim, Eng Wee Chua

**Affiliations:** Faculty of Pharmacy, Universiti Kebangsaan Malaysia, Kuala Lumpur, Malaysia

**Keywords:** inflammatory bowel disease, azathioprine, 6-mercaptopurine, pharmacogenomics, therapeutic drug monitoring

## Abstract

Azathioprine and 6-mercaptopurine, often referred to as *thiopurine compounds*, are commonly used in the management of inflammatory bowel disease. However, patients receiving these drugs are prone to developing adverse drug reactions or therapeutic resistance. Achieving predefined levels of two major thiopurine metabolites, 6-thioguanine nucleotides and 6-methylmercaptopurine, is a long-standing clinical practice in ensuring therapeutic efficacy; however, their correlation with treatment response is sometimes unclear. Various genetic markers have also been used to aid the identification of patients who are thiopurine-sensitive or refractory. The recent discovery of novel Asian-specific DNA variants, namely those in the *NUDT15* gene, and their link to thiopurine toxicity, have led clinicians and scientists to revisit the utility of Caucasian biomarkers for Asian individuals with inflammatory bowel disease. In this review, we explore the limitations associated with the current methods used for therapeutic monitoring of thiopurine metabolites and how the recent discovery of ethnicity-specific genetic markers can complement thiopurine metabolites measurement in formulating a strategy for more accurate prediction of thiopurine response. We also discuss the challenges in thiopurine therapy, alongside the current strategies used in patients with reduced thiopurine response. The review is concluded with suggestions for future work aiming at using a more comprehensive approach to optimize the efficacy of thiopurine compounds in inflammatory bowel disease.

## Introduction

Inflammatory bowel disease (IBD) is an idiopathic, chronic inflammatory disorder caused by dysregulation of the gut immune response (Matricon et al., 2010). It is categorized into Crohn’s disease (CD) and ulcerative colitis (UC), which differ in their anatomical site and pattern of inflammation, and the layers of the gastrointestinal wall that are affected. The etiology of IBD is unclear, but environmental factors appear to trigger immunological hyperactivity in genetically susceptible individuals. Exposure to microbes helps the immune system to establish its repertoire of defensive responses against foreign agents. A hygienic environment presumably provides fewer stimuli for maturing the immune system, thereby lowering immune tolerance. For instance, IBD has been found to be more prevalent in developed countries (Economou and Pappas, 2008). Recently, the gut microbiome has also been linked to the development of IBD, and new IBD therapy such as fecal microbiota transplantation is being explored (Weingarden and Vaughn, 2017).

Corticosteroids, immunomodulators, and biologics comprise the mainstay of IBD treatment. While biologics offer advantages in terms of safety and efficacy, the high cost and the need for parenteral administration limit their clinical usage. Therefore, immunomodulators, particularly thiopurines, are more commonly used in IBD for maintenance therapy. Despite their widespread use, thiopurines are often associated with adverse drug reactions and treatment failures. The variability in response to thiopurine compounds relates to interindividual pharmacokinetic differences. In addition, ethnic differences in the prevalence of certain genetic polymorphisms in the thiopurine-related pathways could affect thiopurine responsiveness (Lee et al., 2015). Understanding this underlying pharmacogenetics of the thiopurine pathway could be useful in helping clinicians to optimize therapy individually for better safety and efficacy. In this review, we will focus on the influences of ethnicity-specific genetic markers on thiopurine response and explore possible mechanisms that underpin thiopurine resistance.

## The Place of Thiopurines in IBD Therapy

Azathioprine and 6-mercaptopurine are the two most commonly used thiopurine compounds in IBD management. Because of their slow onset, requiring at least 12–17 weeks of continuous therapy to produce noticeable effect (Prefontaine et al., 2010), thiopurines have a specific role in IBD, i.e., one of maintaining long-term remission. The recommended dose is 1.5–2.5 mg/kg for azathioprine and 0.75–1.5 mg/kg for 6-mercaptopurine (Gomollón et al., 2017; Harbord et al., 2017). At initial stages of IBD therapy, thiopurines are combined with a short course of a steroid or an anti-TNF agent for rapid induction of remission. Several studies have shown that thiopurines lengthened remission in UC or CD patients. A meta-analysis reported that 73% of CD patients treated with azathioprine were able to maintain remission over a period of 6–18 months, as compared with 62% in a placebo group (RR 1.19, 95% CI, 1.05–1.34) (Chande et al., 2015). Likewise, UC patients given azathioprine were found to have a lower disease relapse rate of 44% as compared with 65% among those treated with a placebo (RR 0.68, 95% CI, 0.54–0.86) (Timmer et al., 2016). However, the strength of the findings presented in both studies is limited by inadequate data and unknown risk of bias (Chande et al., 2015; Timmer et al., 2016). Therefore, the role of azathioprine monotherapy in IBD remains a point of discussion among clinicians. Nonetheless, the European Crohn’s and Colitis Organisation released a consensus statement to support the use of thiopurines as a monotherapy or an adjunct to infliximab in CD and steroid-dependent UC, noting that thiopurines were significantly more effective than aminosalicylates in curbing flares of UC (Gomollón et al., 2017; Harbord et al., 2017).

Thiopurines are also commonly used as adjunct therapy in a step-down strategy for treating severe CD. An intensive drug regimen that includes an anti-TNF agent is used at the beginning of therapy and subsequently de-escalated when remission is attained. In an open randomized trial that investigated the step-down approach, 60% of the patients who received a combination of immunosuppressants were in steroid-free remission compared with 35.9% in a conventional step-up treatment group at week 26 (D’Haens et al., 2008). The Study of Biologic and Immunomodulator Naive Patients in Crohn’s Disease reported similar findings that the infliximab-azathioprine combination was superior to infliximab or azathioprine monotherapy at inducing and maintaining steroid-free remission and mucosal healing (Colombel et al., 2010). Similarly, the UC SUCCESS trial reported that 39.7% of the UC patients receiving the infliximab-azathioprine combination achieved steroid-free remission at week 16, as compared with ∼22–23% patients receiving infliximab or azathioprine alone (Panaccione et al., 2011). The effectiveness of the combination therapy has been ascribed to the protective effect of azathioprine against the formation of antibodies that impede the action of infliximab (Colombel et al., 2010; Panaccione et al., 2014). The beneficial interaction between thiopurines and anti-TNF agents lowers the requirements for the effective drug concentrations and has important implications for therapeutic drug monitoring in IBD, which will be discussed in another section of this review.

## Monitoring Side Effects

Prior to starting thiopurine therapy, a clinical assessment should be performed to ascertain whether patients are at risk of developing opportunistic infection or adverse drug reactions. Patients’ medical and vaccination history is important in establishing their current immunological status. Serological screening for hepatitis B virus, hepatitis C virus, varicella zoster virus, human immunodeficiency virus, and Epstein-Barr virus should be done as recommended by the European Crohn’s and Colitis Organisation. Screening for tuberculosis can be done by chest radiography and tuberculin skin test. Furthermore, vaccination for pneumococcal disease and influenza is required prior to the start of treatment and inactivated trivalent influenza vaccine should be given annually (Rahier et al., 2014). Patients should also be informed that live vaccines are contraindicated once the therapy is started and they should notify their physicians about their condition when getting treatment for other illnesses (Warner et al., 2018).

The common adverse drug reactions associated with thiopurines are leukopenia, hepatotoxicity, pancreatitis, and gastric intolerance (Warner et al., 2018). A full blood count and a liver function test should be conducted before starting a thiopurine and continued every 2 weeks for the first 2 months. If patients respond well to thiopurines, routine full blood counts and liver function tests are recommended every 3 months throughout therapy (Goel et al., 2015). Close monitoring of elderly patients is highly recommended, especially during the first 3 months of therapy as they are at higher risk of developing adverse reactions. A case-controlled study conducted by the Spanish Working Group on Crohn’s Disease and Ulcerative Colitis involving a large cohort of 48,752 adult IBD patients reported that patients older than 60 years receiving thiopurines suffered a significantly higher rate of adverse reactions and treatment discontinuation, compared with those younger than 50 years of age (Calafat et al., 2018).

Another concern over thiopurine therapy is an increased risk of malignancy, specifically lymphoproliferative disorder. Previous studies reported a four- to fivefold increase in the risk of malignancy compared with the general population, although the absolute risk remains very low (Kandiel et al., 2005; Beaugerie et al., 2009). Recently, a large prospective observational study reported a sevenfold increase in the risk of developing a myeloproliferative disorder for those who were previously exposed to thiopurines, but no increased risk for patients who were receiving ongoing thiopurine therapy (Lopez et al., 2014). A rare form of aggressive lymphoma known as hepatosplenic T-cell lymphoma has been associated with the use of thiopurines in CD patients, and the majority of them were young male patients with a median age of 22 years old (Ochenrider et al., 2010). The event rate was so low that it was impossible to estimate the risk; however, regular monitoring is recommended especially for young male CD patients who are currently on or have previously been exposed to a thiopurine. In addition, non-melanomatous skin cancer is another malignancy associated with thiopurine therapy. A systematic review has concluded that thiopurine use in IBD patients was associated with increased risk of non-melanomatous skin cancer with a hazard ratio of 2.1–2.28. However, the authors also cautioned that IBD *per se* is a risk factor for skin cancer, and younger patients were particularly at risk (Hagen and Pugliano-Mauro, 2018). More recently, a large retrospective analysis reported that the rate of malignancy for patients aged > 50 years receiving thiopurine therapy was 18.2%, significantly higher than the rate of 3.8% for patients < 50 years. Treatment duration more than 4 years was also shown to carry a greater risk for malignancy (Beigel et al., 2014). Overall, the consensus is that the benefit of thiopurine therapy outweighs the risk of malignancy or other adverse effects. The findings of those studies provide important information for clinicians to consider when deciding to initiate or withdraw thiopurine treatment. Elderly patients seem notably prone to thiopurine toxicity; therefore, the benefits of thiopurine therapy should be weighed more carefully against its risks for this vulnerable group.

## The Thiopurine Pathway

**FIGURE 1:**
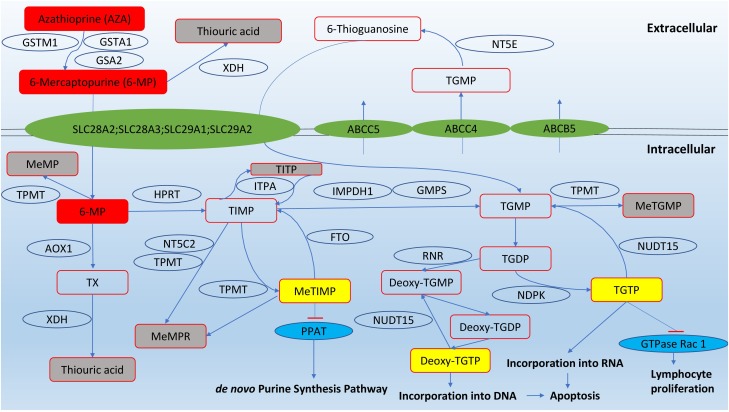
Pathways of thiopurine disposition and action (adapted from Zaza et al., 2010). Cytosolic 5′-nucleotidase II (NT5C2); ecto-5′-nucleotidase (NT5E); fat mass and obesity-associated protein (FTO); nudix hydrolase 15 (NUDT15); glutathione S-transferase mu 1 (GSTM1); glutathione S-transferase alpha 1 (GSTA1); Glutathione S-transferase alpha 2 (GSTA2); thiopurine methyltransferase (TPMT); xanthine dehydrogenase (XDH); hypoxanthine guanine phosphoribosyltransferase (HPRT); inosine triphosphate pyrophosphatase (ITPA); inosine monophosphate dehydrogenase type 1 (IMPDH1); guanosine monophosphate synthetase (GMPS); aldehyde oxidase type 1 (AOX1); phosphoribosyl pyrophosphate amidotransferase (PPAT); ribonucleotide reductase (RNR); nucleotide diphosphate kinase (NDPK); methyl-mercaptopurine (meMP); thioxanthine (TX); methyl-mercaptopurine ribonucleotides (meMPR); thioinosine monophosphate (TIMP); thioinosine triphosphoate (TITP); methyl-thioinosine monophosphate (meTIMP); thioguanine monophosphate (TGMP); methyl-thioguanine monophosphate (meTGMP); thioguanine diphosphate (TGDP); thioguanine triphosphate (TGTP).

The thiopurine compounds are a group of antimetabolites that structurally resemble endogenous purines. Azathioprine is a prodrug that is converted into 6-mercaptopurine by glutathione transferases (GST); alternatively, the conversion can be non-enzymatic. As non-enzymatic conversion accounts for less than 1% of the biotransformation based on an *in vitro* study (Eklund et al., 2006), the role of *GST* polymorphisms was thought to be insignificant in causing variation in azathioprine pharmacokinetics. However, null expression of a highly polymorphic GST subtype, GSTM1, was correlated to reduced azathioprine response in young IBD patients (Stocco et al., 2014). After entry into cells, 6-mercaptopurine is converted by a series of metabolic steps into 6-thioguanine nucleotides (6-TGNs). Other pathways competing with the formation of 6-TGN are methylation and oxidation of 6-mercaptopurine, catalysed by thiopurine *S*-methyltransferase (TPMT) and xanthine dehydrogenase (XDH), respectively (**Figure 1**).

The immunosuppressive effects of thiopurines are produced from three pathways. First, 6-TGN can be converted into deoxy-6-thioguanosine, which causes cell apoptosis by incorporation into DNA and inhibition of DNA-processing enzymes such as topoisomerase and DNA ligase that maintain base-pair stability and DNA dynamics (Somerville et al., 2003). Second, 6-TGNs, in particular thioguanosine triphosphate (TGTP), inhibit the activity of the GTPase Rac1, which regulates T-lymphocyte proliferation, and repress immune responses (Tiede et al., 2003; Poppe et al., 2006). The third route to immunosuppression involves methyl-thioinosine monophsophate (meTIMP), which inhibits phosphoribosyl pyrophosphate amidotransferase, an enzyme that catalyzes the first step of *de novo* purine synthesis (Karim et al., 2013).

## The Role of TPMT Monitoring

The American Gastroenterological Association recommends that TPMT activity should be tested prior to the start of thiopurines and the dose adjusted according to TPMT status (Feuerstein et al., 2017). Owing to its role in methylating and inactivating thiopurines (**Figure 1**), TPMT influences the risk of severe and potentially fatal myelosuppression among patients receiving standard doses of azathioprine or 6-mercaptopurine. Individuals with TPMT deficiency should avoid thiopurine treatment or, if deemed necessary, start with < 10% of the standard initiation dose. Heterozygotes or individuals with intermediate enzyme activity should be given half of the usual dose (Coenen et al., 2015). Deleterious *TPMT* genetic polymorphisms are primarily responsible for the interindividual variability in TPMT enzyme activity. The common alleles causing TPMT deficiency are *TPMT^∗^2, TPMT^∗^3A, TPMT^∗^3B*, and *TPMT^∗^3C* (Salavaggione et al., 2005). The degree of enzyme deficiency depends on whether one or two gene copies are defective. In Caucasians, approximately 10% of the population had at least one of the defective alleles, with *TPMT^∗^3A* being the most common allele. A small proportion (0.3–0.5%) of the Caucasian population are homozygous for the non-wild type allele or completely deficient in TPMT function (Collie-Duguid et al., 1999; Hon et al., 1999). The prevalence of defective *TPMT* alleles varies with ethnicity. As opposed to Caucasians, Asians are much less likely to be TPMT-deficient, with less than 5% of the population having at least one defective allele and almost none of them being a homozygote (Collie-Duguid et al., 1999; Kham et al., 2008). Therefore, TPMT testing in Asians may not be as useful as in Caucasians and it is not included in the routine therapeutic work-up for Asian IBD patients (Lee et al., 2015).

## Thiopurine Metabolites Monitoring

The level of 6-TGN is often measured in clinical studies to gauge the success of thiopurine therapy. To date, much of the work on establishing a therapeutic 6-TGN cut-off has been performed in Caucasian patients. The accepted therapeutic range has stayed around 230–450 pmol/8 × 10^8^ RBC (red blood cell) since its derivation more than a decade ago (Gisbert et al., 2006; Osterman et al., 2006). A popular practice at the clinics is adjusting the dosage and aiming for the target levels to enhance the probability of disease remission in patients with active disease or those who experience side effects (Osterman et al., 2006; Feuerstein et al., 2017; Vande Casteele et al., 2017). However, because of substantial inter- and intra-population differences, defining a threshold 6-TGN level that universally predicts positive clinical outcomes is difficult (Wright et al., 2004; Teml et al., 2007). The observed differences arise from a lack of a standard method for 6-TGN testing, variable study designs, or the intricacies of thiopurine pharmacology that we have yet to understand fully (Konidari et al., 2014).

### Age, Ethnicity, and Anti-TNF Agents

Many studies have documented age- and ethnicity-related differences in the 6-TGN concentrations needed to produce a clinical benefit. A meta-analysis of fifteen studies involving pediatric IBD patients concluded that even though there was positive correlation between clinical outcomes and 6-TGN levels, a definitive therapeutic cut-off could not be ascertained (Konidari et al., 2014). A retrospective cohort study has reported that lower 6-TGN levels of 180–355 pmol/8 × 10^8^ RBC were adequate for Chinese adults to maintain remission (Mao et al., 2017). This coincides with a prior observation that Asians are more prone to thiopurine-induced leukopenia (Lee et al., 2015). These reports suggest that Asians are more sensitive than Caucasians to thiopurines.

The effective 6-TGN levels may also be altered by concurrent drug therapy. The combination of thiopurines with anti-TNF agents is becoming a paradigm for managing IBD. Several benefits of the regimen have emerged from a cross-sectional study involving IBD patients who maintained remission with a combination of infliximab and azathioprine or infliximab alone. The study showed that patients with 6-TGN levels > 125 pmol/8 × 10^8^ RBC had a significantly higher median infliximab trough level than those with 6-TGN levels < 125 pmol/8 × 10^8^ RBC (13.4 mcg/mL vs. 4.3 mcg/mL). These patients also had a higher mucosal healing rate and were less likely to produce anti-infliximab antibodies than those who did not reach the cut-off of 125 pmol/8 × 10^8^ RBC (Yarur et al., 2015). In a more recent study that examined the combination of thiopurines with anti-TNF agents, the action of both infliximab and adalimumab was augmented when 6-TGN levels exceeded 125 pmol/8 × 10^8^ RBC (Kelly et al., 2016). The authors attributed the synergistic effect to the ability of thiopurines to prevent immune responses against infliximab or adalimumab, thus resulting in the higher levels of the two drugs (Colombel et al., 2010; Yarur et al., 2015). A randomized open-label trial [DIAMOND] comparing the effectiveness of adalimumab-azathioprine combination with adalimumab monotherapy found that the combination therapy was significantly more effective than adalimumab alone in achieving the desired endoscopic response at week 26 (84.2% vs. 63.8%); however, the difference diminished at week 52 (79.6% vs. 69.8%) (Matsumoto et al., 2016). Taken together, the findings from the two trials suggest that infliximab-azathioprine may be more suitable than adalimumab-azathioprine in maintaining mucosal healing. The difference in clinical efficacy between infliximab and adalimumab may be due to variation in their pharmacokinetic and pharmacodynamic properties, which can affect the outcome of their interaction with other molecules. Therefore, the benefit observed with the use of infliximab is probably not a class effect, and the clinical benefit of each anti-TNF agent should be individually tested (Fiorino and Danese, 2016).

Aside from the uncertainties about the target range (Reinshagen et al., 2007; González-Lama et al., 2011; Dassopoulos et al., 2014), there is a lack of clear evidence for whether it is beneficial to guide thiopurine dosing based on routine thiopurine metabolites monitoring as compared with conventional weight-based dosing (Feuerstein et al., 2017; Vande Casteele et al., 2017). Therefore, the American Gastroenterological Association does not recommend routine thiopurine metabolites testing. The association, however, conditionally recommends reactive thiopurine metabolite testing in patients experiencing adverse drug reactions or active IBD related symptoms to guide treatment changes, though the quality of evidence is very low (Feuerstein et al., 2017; Vande Casteele et al., 2017). Thiopurine metabolite monitoring is also valuable in detecting non-adherence or underdosage, or identifying patients with aberrant metabolic profiles (Stocco et al., 2010; Gilissen et al., 2012).

**Table 1:** Various genes shown to influence thiopurine responsiveness.

Classification	Candidate genes	Gene variants, changes in gene expression, or phenotype	Outcome	Reference
Thiopurine transport	Influx transporters:			
	i) *SLC28A2, SLC28A3, SLC29A1, SLC29A2*	Downregulation	Thiopurine resistance demonstrated in human cell lines	Fotoohi et al., 2006; Peng et al., 2008; Karim et al., 2011
	Efflux transporters:			
	i) *ABCC5*	Overexpression	Thiopurine resistance demonstrated in human cell lines	Wijnholds et al., 2000; Wielinga et al., 2002
	ii) *ABCC4*	Overexpression	Thiopurine resistance demonstrated in human cell lines	Peng et al., 2008
		rs3765534; rs146708960	Decreased protein expression and increased thiopurine sensitivity	Janke et al., 2008; Krishnamurthy et al., 2008; Ban et al., 2010
	iii) *ABCB5*	rs2031641 G/G	Thiopurine hypermethylation with high 6-MMP levels	Blaker et al., 2012
	Extracellular enzyme:			
	i) *NT5E*	Multiple functional variants affecting expression	Increased expression correlated with enhanced thiopurine sensitivity and higher 6-TGN levels, and vice versa	Li et al., 2010
Thiopurine metabolism	Pro-drug conversion:			
	i) *GSTM1*	Gene deletion; abolished gene expression	Reduced azathioprine therapy response; low 6-TGN/dose ratio for azathioprine	Stocco et al., 2014
	6-TGN synthesis:			
	i) *HPRT1*	Enzyme activity	High enzyme activity correlated with leukopenia and higher 6-TGN levels	Ding et al., 2012
	ii) *IMPDH1*	Enzyme activity	Enzyme activity inversely correlated with meTIMP concentrations; no association with 6-TGN levels	Haglund et al., 2011
		*IMPDH1* P3 Promoter insertion 91–83insGAGCAGTAG	Azathioprine resistance	Roberts et al., 2007
	Inactivation pathway:			
	i) *TPMT*	Enzyme activity	High enzyme activity predicted treatment failures	Ansari et al., 2002; Cuffari et al., 2004
		A complex array of polymorphisms, most notably rs1800462 (*2); rs1800460 (*3B); rs1142345 (*3C); TPMT *3A haplotype that comprises rs1800460 (*3B) and rs1142345 (*3C)	Thiopurine toxicities	Salavaggione et al., 2005
	ii) *AOX1*	rs55754655	Reduced response towards azathioprine; higher requirements for azathioprine dose	Smith et al., 2009; Kurzawski et al., 2012
	Mixed roles:			
	i) *ITPA*	rs1127354	Low enzyme activity; potentially increased 6-TGN levels	Stocco et al., 2009
	Dephosphorylation of active metabolites:			
	i) *NUDT15*	rs116855232	Enzyme deficiency impairing dephosphorylation of TGTP and deoxy-TGTP	Yang et al., 2014; Moriyama et al., 2016
	TGDP to TGTP conversion:			
	i) *NDPK*	Enzyme activity	Reduced enzyme activity could lower TGTP/TGDP ratios; potentially less effective treatment	Karner et al., 2010
Indirect pathway: modulation of enzyme activity	Synthesis of SAM			
	i) *TYMS, MAT1A, MAT2A, MTHFR*	A multitude of variants affecting enzyme activity	The variants could affect SAM production that is important in maintaining TPMT stability; the effect was more pronounced in individuals heterozygous for one of the defective *TPMT* alleles	Karas-Kuzelicki et al., 2010; Milek et al., 2012; Karas-Kuželički et al., 2014
	Molybdenum cofactor activity:	A collection of variants affecting enzyme activity	Significant association with low TPMT activity	Coelho et al., 2016
	i) *MOCOS*		Lowered activity of molybdenum cofactor for XDH; required lower doses of azathioprine (rs594445)	Kurzawski et al., 2012
Others	Endogenous purine synthesis:			
	i) *PRPS1*	Several non-synonymous variants	Reduced feedback inhibition of *de novo* purine synthesis; thiopurine resistance	Li et al., 2015
	Unspecific pathways:			
	i) *FTO*	rs79206939	Reduce FTO protein activity; higher risks of thiopurine-induced leukopenia	Kim et al., 2016
	ii) *PACSIN2*	rs2413739	Altered TPMT activity; increased sensitivity of cells to thiopurines via interaction with Rac1	de Kreuk et al., 2011; Stocco et al., 2012
	Genes identified through profiling:			
	i) *CD1D, CTSS, DEF8, FAM46A, FAM156A, FAR1, GNB4, HVCN1, IMPDH2, LAP3, MAP3K1, PLCB2, SLX1A, SMAP2, TGOLN2, TOX4, TUSC2, UBE2A*	A diverse collection of variants	Correlated well with disease activity and metabolite profiles	Haglund et al., 2013


*6-MMP, 6-methylmercaptopurine; 6-TGN, 6-thioguanine nucleotide; meTIMP, methyl-thioinosine monophosphate; TGDP, thioguanine diphosphate; TGTP, thioguanine triphosphate; SAM, S-adenosyl methionine; TPMT, thiopurine methyltransferase; XDH, xanthine dehydrogenase; FTO, fat mass and obesity-associated.*

### Methods for Measuring Thiopurine Metabolites and Their Limitations

Different methods of 6-TGN measurement can produce discrepant results, thus rendering the findings from many studies not directly comparable (Shipkova et al., 2003; Simsek et al., 2017). For instance, the method reported by Dervieux et al. (2005) gave concentrations that were 2.6-fold those detected by the conventional method (Lennard and Singleton, 1992). The discrepancy may be due to differences in sample preparation and improved detection sensitivity of HPLC technologies. Standardizing the method of 6-TGN measurement seems an obvious solution to the problem, but even the studies which used the same method came to conflicting conclusions. Some studies showed a significant correlation between serum 6-TGN levels and clinical responses, while others showed no relationship. The heterogeneity of these studies in terms of their designs (retrospective vs. prospective), duration of thiopurine treatment, measures of clinical outcome, and differences in baseline patient characteristics may have contributed to the inconsistency (Konidari et al., 2014).

In current clinical practice, thiopurine metabolite measurement is performed using a method which cannot distinguish the mono-, di-, and triphosphates of 6-TGN and meMPR (methyl-mercaptopurine ribonucleotide) (**Figure 1**). TGTP is the predominant phosphate form of thiopurines in RBC and is responsible for their bioactivity (Vikingsson et al., 2009), but measuring all nucleotides together could be useful to provide additional insights into the association between metabolite levels and therapeutic responses. A threshold serum TGTP level of 100 pmol/8 × 10^8^ RBC predicts a positive response to treatment, and patients with an elevated fraction of thioguanosine diphosphate (TGDP) have an attenuated response (Neurath et al., 2005). New methods have been developed to distinguish the three phosphorylated forms of 6-TGN. However, these methods are complex, require extra steps of oxidation, and suffer from relatively low accuracy (50–80%) (Vikingsson et al., 2013) when compared with the conventional method (∼99%) (Lennard and Singleton, 1992).

In most studies published to date, the thiopurine metabolites have been measured in RBCs. However, RBCs are not the site of action of thiopurines. Lacking a nucleus, they do not have all the enzymes required for thiopurine metabolism (Duley and Florin, 2005). This might explain the poor correlation between RBC 6-TGN levels and therapeutic responses as the measurement does not reflect the action of thiopurines in their target sites, i.e., white blood cells and bone marrow. The 6-TGN levels in white blood cells are, however, difficult to measure as the isolation of these cells is often confounded by RBC contamination (Bergan et al., 1997; Duley and Florin, 2005). To overcome this bottleneck, a new method has been developed to quantify the amount of DNA-incorporated 6-TGN, deoxy-6-thioguanosine, by using liquid chromatography-mass spectrometry (Coulthard et al., 2016).

## Genetic Markers of Adverse Effects

### The Novel Asian-Dominant Gene NUDT15

The network of thiopurine metabolism is complex, and the activity of each pathway therein varies across ethnicity (**Table 1**). In this section, we will focus on several genes that have a major influence on the development of thiopurine-related adverse effects in Asians. *NUDT15* is a gene that encodes a purine-specific Nudix hydrolase which is responsible for the hydrolysis of nucleosides-diphosphates. The enzyme is hypothesized to dephosphorylate the thiopurine active metabolites TGTP and deoxy-TGTP, thus hindering the binding of TGTP to Rac1 and incorporation of deoxy-TGTP into DNA (Moriyama et al., 2016).

Unlike other biomarkers discovered thus far, the *NUDT15* variants have been consistently found to have an unfavorable effect on thiopurine metabolism and therefore clinical response. These variants are highly penetrant and sensitive predictors of thiopurine toxicity that are comparable to their *TPMT* counterparts (Yang et al., 2014, 2015). The summation of the influences of individual *NUDT15* variants follows the additive model of genetic inheritance, whereby the severity of the aberrant phenotype is proportional to the number of risk alleles found in the gene (Moriyama et al., 2016). Homozygous carriers of *NUDT15* variants are extremely intolerant of thiopurine compounds and have been shown to be able to tolerate < 10% of a standard dose of mercaptopurine in acute lymphoblastic leukemia patients (Yang et al., 2015). Another study based on *NUDT15*-knockdown cell lines treated with thiopurine compounds showed a significant increase in TGTP levels, with a higher TGTP/TGMP ratio and a higher percentage of TGTP in 6-TGN (Moriyama et al., 2016).

The discovery of the impact of *NUDT15* variants on thiopurine sensitivity marks a significant milestone in the treatment of Asian IBD patients. The most studied marker of thiopurine toxicity to date, TPMT deficiency, has been well established in the Western population; however, *TPMT* mutations are rare and of limited relevance in Asians (Zhang et al., 2004; Gearry and Barclay, 2005; Kham et al., 2008; Lee et al., 2015). Instead, *NUDT15* has now become known as a major genetic determinant of thiopurine response in the Oriental population (Moriyama et al., 2016; Zhang et al., 2017). A non-synonymous variant in the gene, Arg139Cys (rs116855232), is a primary contributor to impaired enzyme activity and thiopurine-related adverse reactions such as myelosuppression and alopecia (Kakuta et al., 2015; Yang et al., 2015; Asada et al., 2016; Chiengthong et al., 2016; Shah et al., 2016; Zhu et al., 2016). The variant is frequently found in East Asians (9.8%) and Hispanics (3.9%), but it is rare in Europeans (0.2%) and absent in Africans (Yang et al., 2015).

Recognizing the risk of excessive immunosuppression and consequently early leukopenia in NUDT15-deficient individuals, the Korean Association for the Study of Intestinal Diseases has recommended that *NUDT15* genotypes should be tested before initiating thiopurine therapy (Lee et al., 2015). However, the cost-effectiveness of the strategy has not been systematically evaluated; so, the recommendation to deploy it at the clinics may have been premature. Furthermore, the interpretation of *NUDT15* genotypes is complicated in patients carrying multiple functional variants, where different heterozygous haplotypes can confer subtle variation in enzyme activity. The current proposal is to reduce the dose of thiopurines for patients carrying deleterious *NUDT15* variants; however, the extent of dosage reduction remains vague, and this has further limited the usefulness of the genotype-guided dosing strategy (Moriyama et al., 2016).

### Beyond NUDT15

The discovery of *NUDT15* has nevertheless rekindled the interest in the influence of ethnicity on thiopurine response. There is a long-standing assumption that Asians are in general less able to tolerate thiopurine drugs; for instance, the average lower doses of azathioprine, ranging from 1 to 2 mg/kg/day, were reported to be sufficient to achieve clinical efficacy and the target 6-TGN levels in Asian IBD patients, as compared with the conventional dosage of 1.5–2.5 mg/kg/day (Andoh et al., 2008; Komiyama et al., 2008; Kim et al., 2009; Lee et al., 2009; Shi et al., 2016). This observation, in tandem with the emergence of *NUDT15*, has quickened the search for other markers that could also help to predict thiopurine efficacy in Asians. Most recently, a genome-wide association study in East Asian IBD patients has discovered a coding variant in the fat mass and obesity-associated (FTO) protein, Ala134Thr or rs79206939. The variant diminishes FTO activity and predisposes individuals harboring the variant to thiopurine-induced leukopenia (Kim et al., 2016). FTO is a member of the AlkB family of Fe(II)/α-ketoglutarate-dependent dioxygenases, which demethylate DNA and RNA (Fedeles et al., 2015). The demethylating action of the FTO protein may serve to counteract meTIMP, a potent inhibitor of purine biosynthesis (Fotoohi et al., 2010), and curb excessive impairment of cell replication. FTO is also responsible for regulating other genes in hematopoiesis, and a reduction in FTO activity can lead to severe myelosuppression (Kim et al., 2016). Interestingly, Ala134Thr is common in Koreans (5.1%) but much less so in Caucasians (< 0.1%) (Kim et al., 2016). In addition, a functional variant of ABCC4 (E857K), an efflux transporter that extrudes thiopurine metabolites from lymphocytes, is prevalent in Japanese IBD patients (>18%) and increases thiopurine sensitivity (Krishnamurthy et al., 2008; Ban et al., 2010).

The effect of variants in other genes seems less clear. A inosine triphosphate pyrophosphatase (ITPA) variant, 94C > A (rs1127354), which is associated with low enzyme activity (Sumi et al., 2002), is more common in Asians (14–19%) than Caucasians (6–7%) (Marsh et al., 2004). ITPAase is involved in the interconversion of thioinosine monophosphate (TIMP) into inactive thioinosine triphosphate (TITP), and low ITPA enzyme activity can increase the 6-TGN levels and the risk of hematological toxicities in acute lymphoblastic leukemia patients (Stocco et al., 2009; Hareedy et al., 2015); however, a meta-analysis of six studies has concluded that ITPA 94C > A was not significantly associated with any of the reported adverse drug reactions (Van Dieren et al., 2007), and more recent studies have shown that low ITPA enzyme activity was not always correlated with side effects from thiopurine therapy (Shipkova et al., 2011; Chiengthong et al., 2016). Furthermore, the role of hypoxanthine-guanine phosphoribosyltransferase (HPRT) may also be ethnicity-specific, as only one clinical report in Chinese IBD patients demonstrated a substantial correlation with thiopurine responsiveness, in contrast to none in Europeans (Ding et al., 2012).

### Thiopurine Hypermethylation and Therapeutic Resistance

Thiopurine compounds are associated with a high rate of non-response, which occurs in approximately 50% of patients given the drugs (Fraser et al., 2002). The resistance to thiopurine therapy can be explained by an individual’s inability to produce sufficient 6-TGNs to achieve therapeutic levels of the active metabolite (Dubinsky et al., 2000; Cuffari et al., 2001; Fraser et al., 2002; Osterman et al., 2006; Moreau et al., 2014).

The 6-TGN under-production in a subgroup of IBD patients is largely believed to be due to thiopurine hypermethylation (Dubinsky et al., 2002). These individuals are noted for their skewed thiopurine metabolism (*shunters*) whereby thiopurine compounds are preferentially metabolized into methyl-mercaptopurine (meMP) and meMPR (**Figure 1**), resulting in inadequate 6-TGN levels and therefore treatment resistance (Dubinsky et al., 2000; Hindorf et al., 2004). A high meMP level of ∼5700 pmol/8 × 10^8^ RBC has been found to cause hepatotoxicity and other thiopurine-induced adverse drug reactions (Dubinsky et al., 2000; Gisbert et al., 2007a; Jharap et al., 2010). Currently, two cut-off 6-TGN/meMP ratios that define hypermethylation have been proposed, i.e., 20:1 (Van Egmond et al., 2012) and 11:1 (Dubinsky et al., 2002; Smith et al., 2012). There is no consensus on which of the two ratios is better at identifying metabolic shunters.

The role of TPMT in inducing thiopurine hypermethylation has been a subject of great interest. It was initially suggested that unusually high activity of TPMT may shift the metabolic machinery toward thiopurine methylation; this has been reported to result in low 6-TGN levels and unfavorable clinical response in IBD and acute lymphoblastic leukemia patients (Lennard et al., 1990; Ansari et al., 2002; Bloomfeld and Onken, 2003; Cuffari et al., 2004). Such enhanced TPMT function has not originated from DNA variation in the gene *per se*, but in those involved in the modulation of TPMT activity. A gene polymorphism in *PACSIN2* (rs2413739) has been noted to increase thiopurine-induced hematological toxicity through, in part, its ability to modulate TPMT activity. Cell lines carrying rs2413739 had higher TPMT activity and were more sensitive to thiopurine compounds (Stocco et al., 2012). *PACSIN2* encodes a lipid-binding protein that interacts with Rac1, the molecular target of thiopurines (de Kreuk et al., 2011). Other genes such as *MOCOS, MAT1A, MAT2A*, and *TYMS*, though not directly involved in thiopurine metabolism, have also been implicated in thiopurine resistance because of their TPMT activity-altering action (Karas-Kuzelicki et al., 2010; Milek et al., 2012; Coelho et al., 2016). However, the hypothesis that TPMT activity is governed by multiple genes has been disproved by findings from a recent genome-wide association study, which has shown that TPMT was a monogenic trait, and that TPMT activity was not affected by non-*TPMT* markers (Liu et al., 2017). Nevertheless, the study has not ruled out regulation of TPMT activity by an unknown epigenomic pathway or factor. Other studies have also countered the utility of measuring TPMT activity for predicting 6-TGN levels and clinical response (Dubinsky et al., 2002; Lennard, 2002; Smith et al., 2012; Van Egmond et al., 2012). The contradicting results from various studies suggest that thiopurine resistance may be a multifactorial phenomenon, in which the exact role of TPMT is clouded by the influences of multiple and possibly obscure pathways.

Our incomplete understanding of the mechanism of thiopurine resistance is worsened by the existence of different phosphate forms of the thiopurine metabolites. A high level of TGTP is desired, as it has a high affinity toward Rac1, and should lead to favorable treatment outcomes (Neurath et al., 2005). The level of TGTP can be increased by nucleotide diphosphate kinase (NDPK), which converts TGDP into TGTP. In theory, variation in the activity of the enzyme could alter TGDP/TGTP ratios and influence thiopurine responsiveness. However, a study of 37 subjects found no correlation between the NDPK activity and the concentrations of the different phosphate forms (Karner et al., 2010). This study, however, is limited by its small sample size. Moreover, high levels of TGDP do not always mean unfavorable outcomes, as the diphosphates can be converted by ribonucleotide reductase into deoxynucleotides, which exert their cytotoxic effect by incorporating into DNA (Zaza et al., 2010).

In addition, meTIMP, which is a precursor of meMPR and conventionally thought to contribute to adverse drug reactions, acts on the purine synthesis pathway and has a different mode of action from TGTP in causing immunosuppression. For instance, in an experiment that used a leukemic cell line, increased formation of meTIMP from TIMP augmented thiopurine sensitivity, but the di- and triphosphates had no effect (Karim et al., 2013). The discovery of the ensemble of phosphorylated thiopurine metabolites provides a possible explanation for the lack of a clear association between TPMT activity and thiopurine resistance. High TPMT activity causes a universal increase in the different phosphorylated forms of the methylated metabolites, which have contrasting actions (Karim et al., 2013); however, they are not routinely discriminated by conventional HPLC detection (Lennard and Singleton, 1992; Vikingsson et al., 2013).

Because of the uncertain role of TPMT and related mechanisms, the involvement of other pathways and enzymes that may affect 6-TGN generation has gained more attention. In some instances, the link of those pathways to thiopurine responsiveness is not immediately apparent. Serving as key enzymes in the conversion of 6-mercaptopurine to 6-TGN, inosine-5′-monophosphate dehydrogenase and HPRT are obvious, though not firmly established, predictors of clinical response (Pieters et al., 1992; Roberts et al., 2007; Haglund et al., 2008; Ding et al., 2012; Moon and Loftus, 2016). Phosphoribosyl pyrophosphate synthetase 1 (PRPS1), whose function is to promote *de novo* purine biosynthesis, can increase the production of endogenous purines, which in turn compete with thiopurine drugs for the same enzymatic pathway, inhibiting their bioactivation (Li et al., 2015).

Another well-known pathway of thiopurine metabolism is the production of inactive metabolites thiouric acid, which involves XDH and aldehyde oxidase (AOX). 6-Mercaptopurine is converted to thiouric acid through sequential metabolism of thioxanthine intermediates involving both AOX and XDH (Choughule et al., 2014). A cohort study of 192 IBD patients has reported that the presence of an AOX1 variant rs55754655 together with high TPMT activity predicted a lower chance of patients responding to azathioprine treatment (Smith et al., 2009). This observation is indirectly supported by another study involving kidney transplant recipients, where the carriers of rs55754655 required a higher dose of azathioprine to maintain immunosuppressive effect at 3, 6, and 12 months after transplantation (Kurzawski et al., 2012).

The transmembrane transporters have also been shown to regulate the intracellular levels of 6-TGN. The down-regulation of influx transporters of 6-mercaptopurine including SLC28A2 (CNT2), SLC28A3 (CNT3), SLC29A1 (ENT1) and SLC29A2 (ENT2) has been shown to render lymphocyte-derived cell-lines resistant to thiopurines, owing to decreased uptake of thiopurine metabolites into the cells (Fotoohi et al., 2006; Peng et al., 2008; Karim et al., 2011). In addition, increased activity of efflux proteins has been associated with thiopurine resistance. ABCC4 and ABCC5 are the two transporters responsible for the efflux of thiopurine intermediates and metabolites (Wijnholds et al., 2000; Wielinga et al., 2002; Peng et al., 2008; Smith et al., 2010). The cells that overexpressed the transporters have been found to contain low levels of 6-TGN and other thiopurine metabolites (Krishnamurthy et al., 2008). The efflux mechanism is counteracted by *thiopurine cellular circulation*, whereby thiopurine nucleotides extruded by ABCC4 will be salvaged by an extracellular enzyme NT5E through hydrolysis into nucleosides, before being transported back into the cells by nucleoside transporters (Li et al., 2010). Several other subtypes of the efflux transports also exist. The action of each transporter is substrate-specific, giving rise to their differing roles in regulating the meMP/6-TGN ratio intracellularly. For instance, inactivating polymorphisms of ABCB5 have been found to cause accumulation of the methylated metabolites but not 6-TGNs inside cells (Blaker et al., 2012).

Finally, several genetic markers and enzymes have been reported to cause thiopurine resistance, but the mechanisms are obscure. Variants in the intronic or flanking regions of the genes directly involved in the thiopurine pathway were correlated with unfavorable treatment response, but the functional impact of the variants was unclear (Matimba et al., 2014). Even less clear is the significance of non-thiopurine pathway-related genetic markers, which have been associated with poor outcomes of thiopurine treatment (Haglund et al., 2013; Matimba et al., 2014).

## Strategies to Overcome Thiopurine Resistance

### Dose-Splitting Regimen

A novel dose-splitting strategy has been used to correct unfavorable response to thiopurine therapy in lieu of the conventional weight-based dosing approach. Dividing the total daily dose of azathioprine or 6-mercaptopurine in thiopurine-resistant patients has been shown to reduce meMP levels and the development of adverse drug reactions, without compromising the levels of 6-TGNs and the clinical efficacy of thiopurines (Shih et al., 2012). This approach not only eliminates the need for the addition of allopurinol, it may even allow further upward titration of thiopurine doses to achieve the desired efficacy with little to no risk of adverse drug reactions (Bradford and Shih, 2011). The proposed rationale of the split-dose strategy is that the reduced dose of thiopurines in each administration produces suboptimal substrate affinity for TPMT, which prevents induction of TPMT activity during treatment (Weyer et al., 2001).

### Thiopurine-Allopurinol Combination Therapy

The current clinical practice in managing thiopurine shunters is through combining low-dose azathioprine or 6-mercaptopurine with allopurinol. The thiopurine dose is reduced by 25–50%, and 100 mg of allopurinol is added (Amin et al., 2015; Fong et al., 2015), though a lower dose of allopurinol has been suggested to be equally efficacious yet safer, with a lower risk of leukopenia and opportunistic infection (Ansari et al., 2008; Govani and Higgins, 2010; Curkovic et al., 2013). Several studies that tested the use of the strategy in IBD patients have reported a significant reduction of the meMP concentration alongside an increase in the 6-TGN level (Sparrow et al., 2005; Ansari et al., 2010; Appell et al., 2013). In the assessment of clinical outcomes, the combination therapy has proven to be safe and efficacious in maintaining long-term steroid-free remission, with improved IBD disease activity scores in thiopurine shunters (Sparrow et al., 2007; Leung et al., 2009). Also, the combination therapy may be especially valuable to patients with extreme thiopurine sensitivity, resolving hepatotoxicity that some patients experienced during the conventional thiopurine monotherapy (Smith et al., 2012; Beswick et al., 2014; Johnson et al., 2014).

The mechanism of allopurinol in optimizing thiopurine therapy is complex and may lie in the ability of allopurinol to inhibit XDH and saturate or limit the methylating capacity of TPMT (Elion, 1989). XDH is involved in the conversion of 6-mercaptopurine to inactive thiouric acid (**Figure 1**). The inhibition of this auxiliary pathway by allopurinol should allow a larger fraction of 6-mercaptopurine to be converted into 6-TGN and meMP, although two studies documented a contradictory decrease in the meMP levels (Sasaki et al., 1987; Oláh et al., 1994). In human RBCs, allopurinol is successively converted by several enzymes to oxypurinol riboside monophosphate, which is a 6-oxo analog of 6-TIMP and a substrate of TPMT (Duley et al., 1985, 2005). Direct TPMT inhibition by allopurinol or oxypurinol has not been demonstrated *in vitro* (Sparrow et al., 2005, 2007). Instead, it has been proposed that allopurinol increases the production of thioxanthine, which acts as a TPMT inhibitor (Blaker et al., 2013).

Allopurinol may also directly heighten 6-TGN production. A prospective study involving IBD patients with aberrant metabolic profiles recorded an increase in HPRT activity following the thiopurine-allopurinol therapy but no effect on TPMT activity, suggesting that allopurinol may affect HPRT through an unknown mechanism (Seinen et al., 2013). Other as-yet undiscovered enzyme cofactors, targeted by allopurinol, may also play a role in thiopurine methylation (Leong et al., 2008).

### Dose-Splitting Regimen or Thiopurine-Allopurinol Combination Therapy?

There is at present a lack of evidence to show which option is superior, except for one small cohort study in IBD pediatric patients. The study reported that patients treated with a combination of thiopurine and allopurinol were more likely to achieve the desired 6-TGN levels than those receiving the split-dose regimen (Chadokufa et al., 2016). However, the study is limited by a small cohort size and its focus on a pediatric population, whose pharmacokinetic profile differs from that of adult patients. In clinical practice, the choice of a treatment strategy for IBD patients is based on other factors including medical history, economic status, and therapeutic compliance.

## Other Factors Influencing Thiopurine Treatment Outcome

Before prescribing thiopurines to the patients, several factors should be considered, as they can affect the outcome of thiopurine therapy. Thiopurines should be used with caution in smokers, as long-term smoking can upregulate CYP450 that can cause demethylation of meMP, potentially leading to a greater proportion of the drug being converted to 6-TGN, though this has not been clinically proven (Warner et al., 2016). On the other hand, excessive alcohol consumption during thiopurine treatment has been reported to cause peliosis hepatis (Elsing et al., 2007), as hepatotoxicity is a common adverse effect of both alcohol and thiopurines. Furthermore, long-term consumption of alcohol could disrupt the methionine cycle, causing depletion of S-adenosylmethionine (Martinez-Chantar et al., 2002), which is essential to the catalytic activity of TPMT (Arenas et al., 2005). This may then predispose patients to thiopurine-induced toxicity, as 6-TGN levels may rise to a dangerous level. TPMT inhibition was also observed in patients who took a combination of a loop diuretic and a thiopurine (Lysaa et al., 1996). Similarly, increased 6-TGN and a higher rate of thiopurine intolerance were reported with the mesalamine-thiopurine combination therapy (Lees et al., 2008; Gao et al., 2012), and the withdrawal of mesalamine caused a drop in 6-TGN levels (Dewit et al., 2002; Gisbert et al., 2007b; Lees et al., 2008). Finally, in elderly patients, thiopurines are not an ideal choice of therapy, as the benefits of taking thiopurines for more than 5 years may not outweigh the risks (Lewis et al., 2000). This is due to the dysregulated immune function in elderly patients, leading to their increased susceptibility to infection, malignancies, and other adverse drug reactions (Gavazzi and Krause, 2002; Weng, 2006; Castelo-Branco and Soveral, 2013).

## Concluding Remarks and Suggestions for Future Work

We have not fully grasped the complexity of thiopurine pharmacology. Asians and Caucasians differ subtly in how they metabolize thiopurines. Through pharmacogenetic investigations, two major pharmacokinetic markers have emerged. Inactivating *NUDT15* variants impair the breakdown of Rac1-binding TGTPs and sensitize Asians to thiopurine toxicity (Moriyama et al., 2016). In Caucasians, *TPMT* variants resulting in low TPMT activity skews the thiopurine pathway to produce excessive 6-TGNs (Gearry and Barclay, 2005). Complete TPMT deficiency, for which TPMT genotyping would be most useful, is rare and irrelevant in Asians (Zhang et al., 2004; Kham et al., 2008).

Together, the two diverging routes of metabolism are gatekeepers of thiopurine toxicity. At the clinic, *NUDT15* or *TPMT* screening can inform initial decisions on treatment eligibility or drug dosages. Further fine-tuning of the treatment regime can then be guided by metabolite profiling. Such a tiered approach may reduce the development of costly adverse drug reactions, while preserving drug efficacy. However, the dosage recommendations for patients with NUDT15 deficiency are lacking, possibly because ascertaining the exact relation between *NUDT15* alleles and 6-TGN levels is challenging. When NUDT15 function is diminished, the resultant accumulation of TGTP is offset by a decrease in TGMP. The net outcome is that 6-TGN levels remain apparently unchanged (Moriyama et al., 2016).

Quantifying TGTPs and TGDPs by liquid chromatography is imprecise, owing to rapid interconversion of the two metabolites (Vikingsson et al., 2013). In routine metabolite testing, TGMP, TGDP, and TGTP are reported collectively as ‘6-TGNs’. Basing clinical decisions on 6-TGN levels alone can overlook NUDT15-deficient patients who are prone to thiopurine-induced myelosuppression. In these patients, 6-TGN levels may appear normal despite elevated TGTP (Moriyama et al., 2016).

Overall, surveys of thiopurine-related genes revealed that nucleoside phosphorylation has a noteworthy role in governing thiopurine sensitivity. The number of phosphate groups attached to thiopurine metabolites dictates their action, and only TGTP, dexoy-TGTP and meTIMP are bioactive. This may explain why some patients do not respond to optimal levels of 6-TGNs or suffer adverse effects from high levels of meMPs (Wright et al., 2004; Teml et al., 2007). A method needs to be devised with adequate resolving power for distinguishing the phosphorylated metabolites in blood samples. Then, we can revisit in detail how changes in NUDT15 or TPMT activity influence metabolite levels and drug efficacy, and possibly reset therapeutic thresholds. It is worth noting that no study has been conducted to ascertain the connection between the extent of *in vivo* 6-TGN and meMPR phosphorylation and thiopurine sensitivity in Asian IBD patients. Comparing the variation in the levels of these metabolites or the activity of enzymes such as NDPK between Asian and Caucasian patients may uncover new findings for optimizing the use of thiopurines across ethnic groups.

Prior genetic research has shed light on the possible origins of excess methylated thiopurine derivatives, i.e., altered cellular transport of the metabolites, impaired 6-TGN synthesis, and alternate routes of metabolism, driven by TPMT or other enzymes, that deviate from 6-TGN production (Fong et al., 2015). Pre-emptive multigene testing may help to identify patients who are unlikely to respond well to a thiopurine and therefore better suited for a biologic or the thiopurine-allopurinol regimen. We envision that the use of thiopurines would become increasingly selective to lower the likelihood of adverse effects, and the process of therapeutic drug monitoring in IBD would be refined further. For instance, the combined action of a thiopurine and an anti-TNF agent may change the definitions of therapeutic 6-TGN levels.

Future work should also focus on the pharmacodynamic aspects of thiopurines. With the advancement of high-throughput sequencing technologies, whole-genome or -exome sequencing has made it possible to examine potential factors that can affect the thiopurine pathway. We surmise that the causative factors may lie within the overarching Rac1 networks, which regulate cell proliferation. Finally, through a multipronged approach that enhances the efficacy of thiopurines, the place of the drugs should continue to remain firm in IBD management.

## Author Contributions

EWC conceived the outline of the manuscript and reviewed and edited the initial and final drafts of the manuscript. SZL wrote the manuscript.

## Conflict of Interest Statement

The authors declare that the research was conducted in the absence of any commercial or financial relationships that could be construed as a potential conflict of interest.
